# User-determined end of net life in Senegal: a qualitative assessment of decision-making related to the retirement of expired nets

**DOI:** 10.1186/1475-2875-12-337

**Published:** 2013-09-22

**Authors:** Dana K Loll, Sara Berthe, Sylvain Landry Faye, Issa Wone, Hannah Koenker, Bethany Arnold, Rachel Weber

**Affiliations:** 1 Center for Communication Programs, Johns Hopkins Bloomberg School of Public Health, Baltimore, MD, USA; 2 Department of Sociology, University Cheikh Anta DIOP, Dakar, Senegal; 3 Department of Public Health, University Cheikh Anta DIOP, Dakar, Senegal

**Keywords:** Malaria, Senegal, LLIN, ITN, Bed nets, Qualitative, Net life span

## Abstract

**Background:**

Procurement and distribution of long-lasting insecticidal nets (LLINs) in the African region has decreased from 145 million in 2010 to 66 million nets in 2012. As resources for LLIN distribution appear to stagnate, it is important to understand the users’ perception of the life span of a net and at what point and why they stop using it. In order to get the most value out of distributed nets and to ensure that they are used for as long as possible, programmes must communicate to users about how to assess useful net life and how to extend it.

**Methods:**

Data were collected from 114 respondents who participated in 56 in-depth interviews (IDIs) and eight focus group discussions (FGDs) in August 2012 in eight regions in Senegal. Households were eligible for the study if they owned at least one net and had an available household member over the age of 18. Data were coded by a team of four coders in ATLAS.ti using a primarily deductive approach.

**Results:**

Respondents reported assessing useful net life using the following criteria: the age of net, the number and size of holes and the presence of mosquitoes in the net at night. If they had the means to do so, many respondents preferred the acquisition of a new net rather than the continued use of a very torn net. However, respondents would preferentially use newer nets, saving older, but useable nets for the future or sharing them with family or friends. Participants reported observing alternative uses of nets, primarily for nets that were considered expired.

**Conclusions:**

The results indicate that decisions regarding the end of net life vary among community members in Senegal, but are primarily related to net integrity. Additional research is needed into user-determined end of net life as well as care and repair behaviours, which could extend useful net life. The results from this study and from future research on this topic should be used to understand current behaviours and develop communication programmes to prolong the useful life of nets.

## Background

Despite advances in the fight against the disease, malaria remains a serious threat to the health and well being of populations in endemic countries. The use of long-lasting insecticidal nets (LLINs) has been proven to reduce contact between the vector and humans, thereby reducing transmission of the disease [1] and LLINs have become an essential component of malaria control programmes worldwide [2]. Between 2004 and 2010, the number of insecticide-treated nets (ITNs) distributed in sub-Saharan Africa increased rapidly from six million to 145 million nets and the percentage of households with at least one net rose to 53% [2]. This scale-up of ITNs in combination with other key interventions was responsible for the aversion of 52% of malaria cases and 58% of malaria-related deaths over the same period [2].

However, LLINs have a limited lifespan, developing tears and holes and losing insecticide over time; field studies indicate that the useful life of a net may vary between 18 months and seven years, with an average expected lifespan of three years, as defined by the World Health Organization (WHO) [3,4]. While other studies focus on the physical integrity and durability of the net, it is the household that ultimately decides when their net is no longer useful. While there is no clear universally accepted definition of the “useful life of a net,” this study defines the term as a mosquito net that is meant to be slept under for malaria prevention and provides a certain level of protection. This paper reports on useful net life largely from the net user’s perspective. The perception that a net is still useful can be understood as it having an active status while the view that it is no longer useful denotes an expired status. This study explores user perceptions of net end of life in order to inform communication interventions to prolong the amount of time that they are deemed to be useful.

Since mass campaign distribution of LLINs have been successful in increasing net ownership [5-7], research on net use and net non-use has become increasingly important. Existing research has documented barriers to the use of nets, which include misconceptions of malaria symptoms and transmission, perceptions of low malaria risk, perceptions of net ineffectiveness, issues related to discomfort, structural inconveniences, use of nets for other purposes, and other social factors [8-23]. While these barriers to daily use of nets are well characterized, the cues to the household determination that nets have reached ‘expired’ status and are no longer useful are not well documented. Since donor funding has stagnated and the number of nets procured has decreased since 2010 [2,24,25], nets will need to be used for longer periods of time to ensure that users are reaching the minimum of three years use and therefore to ensure that universal coverage calculations remain effective. Few studies have considered factors contributing to the user-determined end of life, particularly as nets age and deteriorate.

User-determined end of life appears to be highly variable and complex but very little research has been conducted on when people stop using their nets. Existing research suggests that cues to discarding a net are primarily tied to the damage to the net but can also include its age and the availability of other nets [15,26]. In some contexts even nets received during the previous year are discarded because of the holes that develop [15]. Ngondi and colleagues [27] showed that a decline in the proportion of nets being used over time was independently associated with increasing net age and increasing damage to nets. The poor condition of nets may serve as a barrier to use, especially if the owner perceives that they may no longer be effective [15,16]. This extensive damage can occur as little as three to four months from receipt of the net [16]. For this reason, Pulford and colleagues [8] discuss the need to distinguish between barriers to use of ‘active’ nets with perceived useful life and ‘expired’ mosquito nets, or nets that are deemed no longer useful for malaria prevention.

Current evidence shows that expired nets are sometimes repurposed, saved, or given to a family member or friend rather than being discarded [22,26,28]. Observational data in Tanzania showed that about 16% of expired nets were used for purposes other than preventing malaria such as curtains or for protecting chicks [26]. Thus far, research the feasibility of collecting expired nets for disposal or recycling has shown that these net collection or exchange programmes are unlikely to be successful due to the perceived high economic value to the household of the net or its fabric for sleeping and for other uses, and due to the financial costs of locating and transporting expired nets [28,29].

In a 2009 post-campaign survey conducted in regions of Senegal that had received an integrated campaign targeting children under 5 years, researchers found that 60% of all households had a net that they no longer used, either because they were too old (26%) or had too many holes (28%) [30]. It is therefore important to gain insight into the distinction between ‘active’ and ‘expired’ nets in Senegal, and the way that this affects net use and net coverage. The user perception of this distinction is not yet well understood or documented in the literature. It is the intent of this article to describe the user perceptions of end of useful net life and the cues that cause users to retire a net. The article will explore what is done with these ‘expired nets’.

## Methods

### Study sites

This research was conducted in Senegal, during the period of the country’s first national level universal coverage campaign. Between May 2010 and June 2012, the Senegal National Malaria Control Programme and partners engaged in a universal coverage campaign using a strategy providing households with one net for each sleeping space. The campaign was implemented in five phases across 12 regions of Senegal and resulted in the distribution of 4,070,976 nets [31]. The universal coverage campaign was completed in the remaining two regions in Senegal between January and April, 2013. This study collected data in eight regions of Senegal in order to allow for analysis of variations due to geographical and cultural differences, as well as differences between regions that had benefitted from the universal coverage campaign at different stages. Table 1 provides an overview of the timing of the universal coverage activities in the study regions. Within each region, a rural and a peri-urban site were selected for data collection.

**Table 1:** Timing of universal coverage campaigns in study regions

	**Phase 1**	**Phase 3**	**Phase 4**	**Phase 5**	**Phase 6**
**Regions of intervention**	Kédougou Kolda	Fatick	Saint Louis	Louga Ziguinchor	Thies Dakar
**Time period**	May – Oct 2010	Apr – July 2011	Sep – Dec 2011	April – June 2012	Jan – April 2013

### Study population and procedures

The data were collected during the second phase of a two-phase primarily qualitative study. The first phase focused on general topics including perceived causes of malaria, value of nets, perceptions of nets and net allocation in contexts of insufficient nets. Findings from the first phase, conducted in January 2012, informed discussion topics for the second phase. Questions about end of net life were added to the second phase of the study and therefore, the analysis and results presented in this paper focus on the second phase of research.

Data were simultaneously collected 21–31 August, 2012, during the height of the rainy season, by four data collection teams, each responsible for two regions. Sixteen researchers were responsible for data collection and had been trained on the study objectives, study design and ethical treatment of human subjects.

Participants in the IDIs and FGDs were eligible for the study if their compound owned at least one net and if they were over the age of 18. Once the eligibility criteria were verified, the head of compound and the IDI participant were asked to provide consent to participate. There were eleven refusals to participate in the study; seven were among IDI participants and four were among FGD participants. Refusals were more common in peri-urban areas, where work schedules did not allow participants to commit to the time necessary to complete the research. IDIs were conducted with a randomly selected member of the household in order to maximize the perspectives attained in the study. During the FGDs and IDIs, respondents were presented with two fictitious, projective scenarios in order to understand their decision-making process related to the retirement of nets. First, respondents were shown an old, torn net and were asked about their perceptions of the net. Nets were of variable ages and were retrieved from the homes of the data collectors and torn in a similar way, according to the discretion of the study team. They were asked to reflect on what they would do if the net belonged to them and the reasons for this decision. In the second scenario, participants were told to imagine that they had a one-year-old net that had only a few small holes. They were then asked what they would do if they received a new net.

Focus groups were conducted in four of the eight regions and participants were purposively sampled from the selected communities based on age, sex and net ownership. Focus groups relied on a hybrid guide of phase one and two areas of interest and were therefore conducted only in regions newly entering the study. IDIs, however, utilized a completely new interview guide and were conducted in all eight regions. Focus group discussions were homogenous by sex and ranged in size from six to nine participants. The total sample size for the study was 114 participants including 56 IDI respondents and 58 focus group participants, representing eight FGDs. This total sample included perspectives from 54 women and 58 men. In two IDIs, the sex of the respondents was not recorded. Of the 56 households in the study, 24 had previously been visited in the first phase.

### Data analysis

Interviews and discussions were conducted and audio-recorded in local languages including Wolof, Serere and Pulaar, and then transcribed and translated verbatim into French in Microsoft Word. These textual data were entered into ATLAS.ti and coded by a team of four independent coders using a codebook of themes of interest. Coding was conducted using a primarily deductive approach while allowing for the addition of emergent codes and themes. The coders met frequently throughout the coding, to ensure that codes were being applied in the same way and to discuss the addition of codes as themes arose from the data. The analysis focused on overall perspectives and regional variations regarding end of net life.

### Ethical considerations

Ethical approval for each phase of research was secured from the Johns Hopkins University Bloomberg School of Public Health Institutional Review Board in Baltimore, Maryland, USA and from the Comité National d’Ethique pour la Recherche en Santé in Dakar, Senegal. Participants provided oral consent prior to participating in the study.

## Results

### When is a net no longer useful?

The condition of the net and the availability of other nets were the primary considerations when determining that a net was no longer useful for protection against malaria. Most respondents said that a net was no longer useful when it had many small holes or a few large holes in it. They noted that the presence of the holes themselves made a net unsuitable for sleeping under since the holes increased the risk of human-mosquito contact and malaria. Others said that the presence of mosquitoes in the net due to the holes and the resulting inability to sleep was a trigger for the non-use of the net. These responses were the most common across all study sites and did not vary regionally. However, some respondents discussed the actual age of the net as a determining factor and reported that nets should be replaced at one, one and a half, three, or five years of age.

At least when it has three years and it starts to have tears, you’ll know that it’s old. Sometimes it can last even longer than three years ; nets last a long time.

Ziguinchor urban (Djiringho) Male IDI

Some respondents mentioned that the useful life of a net is largely dependent on care of the net. One respondent noted that nets last longer when not washed with Omo (powder detergent) or bleach. Some participants stated that access to a net of higher quality was a factor that affected their decision to stop using an older net. One respondent noted that he thought the distribution happened only when it was appropriate to change one’s nets.

You must wait and see that the net is damaged and those who distribute the nets know better than we do at what point a net begins to deteriorate. That’s why they replace the nets. Those who replace the nets know at what point a net begins to deteriorate. If they bring in other (nets), it’s that they know that those that they distributed, have begun to go bad. It’s not a coincidence.

Fatick rural (Pakala) Male IDI

While there was no definitive answer from respondents about the when nets expire, most respondents agreed that nets are active for a maximum of five years and their activeness is largely dependent on the care of the net.

### Scenario One: what is done with old, torn nets?

To understand how respondents make decisions about what to do with old or torn nets, participants were asked hypothetical questions about an old, torn net. To help the respondents visualize the net, respondents were shown a used net that had been torn by the study team. To make responses more comparable and aid in interpretation, the nets used in all study sites were comparably torn. Most respondents indicated that the net was no longer useful and that they would prefer to get a new net if they had the means to do so.

Respondent 4 *: If you hang a net and you realize that it has begun to wear out, you should not try to sew it, nor give it to a tailor, nor attempt to modify it, you need to throw it in the trash and then search for another to protect you because you cannot do anything if you are not in good health.*

Respondent 7 *: I agree; this net is useless because it is fragile*.

Thies urban (Pout) FGD females

Due to the increasing availability of nets through universal coverage campaigns and continuous distribution programmes, respondents felt that they were more likely to have the means of getting a new net than in the past. They mentioned the increased number of nets in their communities among friends and family members as well as increased access to nets through distributions. However, many of the respondents mentioned that it would be difficult to obtain a new net due to lack of financial resources. Some of these respondents stated that they would still discard the torn net and thought that it would provide better protection against mosquitoes if they slept with mosquito coils or other malaria prevention methods rather than under the old, torn net.

Respondent: *I would not use it anymore; I will find another or use another method, some method that could at least protect you from malaria.*

Interviewer: *What are these methods?*

Respondent: *Apart from the mosquito net, there is the mosquito coil, Yotox spray [insecticidal spray], and many others. The spray, the coils that you light so that the smoke repels the mosquitos. I prefer these methods to the net because considering this net, it would not do anything for me.*

Dakar rural (Deni Malick Gueye) Male IDI

Few respondents said that they would attempt to repair the old, torn net; repair was seen as a temporary solution to postpone the inevitable need to discard the net. A small minority of respondents stated that they would continue to use the torn net until a new net in better condition became available.

### Scenario Two: what to do if you have a useful net and receive a new one?

In the second hypothetical scenario, participants were asked to describe what they would do if they had a one-year-old net with a few small holes in it and then received a new net. The most common response from all participants was to start using the new net because it was more effective than the one-year-old net. In Ziguinchor and Louga, where the mass campaign had most recently distributed nets, differing opinions were found. In Ziguinchor, while some participants said that they would discard the one-year-old net, the majority stated that they would either save the older net for the time when the new net became torn or give the older net to a neighbour or family member to use.

Respondent: *Well, I will use the new one and keep the old one that has holes in a few places because the new one has the insecticide in it while the old one has lost its insecticide and it also has holes. The new one that will last longer, and I will keep the old one.*

Interviewer: *Now, the old one that you will still keep, what is its usefulness? What will you do with it?*

Respondent: *I’ll find someone who does not have a mosquito net and give it to him then tell him to repair the holes.*

Ziguinchor rural (Coubalan) Male IDI

In contrast, in Louga where nets had been distributed most recently, the majority of respondents stated that they would continue to use the older net and save the new net for a time when the old net was no longer useable.

Respondent: *Like this, the nets will last a long time. If you have a new one and the old one is still intact, I can keep using it until it wears out, after that I’ll use the other one.* Louga urban (Mbassine) Male IDI

Decisions were context specific, depending on the availability and allocation of nets within the respondents’ households and in their communities. Some participants stated that they would need to assess others’ need for nets before making a decision. In Kolda, one female IDI respondent stated:

I will take down the old net, wash it first then keep it someplace. I cannot throw it out because someone else may have a need for it. Or, if the children need it, I can give them the net. I even have another choice: I can give the new net to the children and I take the old net. I know how to avoid mosquitoes so I can use the old net but the children are more vulnerable and need the new one.

Interviewer: *So you take the old net and you give the new one to the children. Madame, why this choice?*

Respondent: *I would make this choice because I am older than the children. I’ve lived and malaria cannot hurt me. But the children have just begun to live their lives and much remains before them still.*

Kolda rural (Guire Yero Bocar) Female IDI

### Alternative uses of nets: how and when?

Respondents were asked about how and when people use nets for purposes other than sleeping, and typically described what other community members did with their nets rather than their own experiences. Respondents felt that torn nets, no longer useful for protection from malaria while sleeping, could be repurposed. They stated that it was rare to find people in the community using brand new nets for alternative uses such as protecting plants.

Respondent: *Here, in general, once the nets have been used up, we bring them to the fields because we do not want to leave them in the house to clutter things. We have seedlings and there are things that will destroy them so we protect them by covering them with old nets.*

Interviewer: *If you bring the nets there, you cover the plants with them?*

Respondent: *No, if you first plant the seedlings, they must be covered. You should leave them covered until it’s time to harvest.*

St Louis rural (Fanaye) Male IDI

Alternative uses of nets fell into two categories: insecticide-dependent uses and insecticide-independent uses. Insecticide-dependent uses are those that relied on the presence of insecticide to repel insects and included using nets as curtains in windows, covering oneself while watching television outdoors, and using as a blanket for protection against fleas. Insecticide-independent uses included using nets for fencing for livestock, covering tombs, preparing couscous, fishing, stuffing pillows, filtration of water and coffee, and as a wash cloth. Alternative uses that can be categorized as both insecticide-dependent and/or independent include covering meat and food for sale at market stalls to protect from insects and protecting seedlings or gardens. In these cases, it was unclear whether the physical barrier of the net or the insecticide itself was more important in the alternative use. Some respondents felt that instead of being repurposed, old nets should be repaired and given to someone without a net; some felt that the insecticide on nets made them dangerous to use for other things like filtering coffee or steaming couscous. Respondents in Dakar, Thies and Kedougou were unable to describe any alternative uses of nets and indicated that this was not practiced in their communities.

## Discussion

The results from this study show that variable criteria, mostly related to the visible condition of the net, are used by households to determine that a net has reached expired status. These criteria included the age of the net, the number and size of holes in the net, the presence of mosquitoes in the net and the availability of other nets within the household or community that could replace the net in question. This finding on availability of other nets and required resources to obtain additional nets is consistent with results in other settings that suggest that poverty and availability of disposible income affect decisions related to net use and retirement [29]. The results also show that the receipt of a new net is not likely to itself serve as a cue to retiring a net. Upon receipt of a new net, somewhat old but still useful nets were likely to be shared with others or saved until a new net became worn. Respondents in Louga were more likely to report that they would continue to use a somewhat old net and save the new net for a future point in time. In addition, this work shows that when nets are very torn, participants preferred to get a new net if they are able. In this case, torn nets can be given away to others, thrown away or repurposed through alternative uses. The results of this study, while specific to communities in Senegal, provided insights into user-determined end of net life that are consistent with and build upon existing literature.

While the results showed a number of cues to retiring a net, most of the criteria were related to the condition and integrity of the net. Respondents reported that they would often use a net until they perceived it to be irreparably damaged and determined that it had expired due to the presence of holes in the net or the mosquitoes in the net at night due to the holes. This is consistent with other research, which states that the condition of the net often determines whether the net is used [8,15,26]. However, Pulford and colleagues [8] showed that the perceived loss of net effectiveness due to diminishing insecticide was seen as a barrier to use. The results of this study did not show perception of reduced insecticide efficacy to be a major factor for determining end of net life in Senegal.

Since the integrity of the net is the most salient criteria for determining whether a net is useful, the way that a net is cared for and repaired becomes increasingly important for preventing and repairing damage. Existing research has shown that net care and repair behaviours are likely to impact net longevity and durability by prolonging the protective lifespan of LLINs [32,33]. For example, frequent washing of LLINs has been shown to result in decreased integrity and insecticide effectiveness [32,33]. In addition, these results are consistent with existing research that net repair is relatively rare [22,32]. In order to extend useful net life, net distribution programmes should provide information on net care and repair practices to maintain net integrity. Existing research has provided evidence of successful strategies for promotion of net care and repair [34-36]. In the Gambia, where nets were not being repaired due to competing demands for time, the promotion of net repair through song was used as a culturally compelling mechanism and resulted in a significant increase in net repairs [34]. In Peru, small-scale trials of improved practices (TIPS) were used to change net care and repair behaviour in accordance with local capacity [35]. The results of this research showed that by involving members of the community, they were able to increase net storage practices, including taking the net down, folding it and storing it every day, and lengthen the time between washings [35]. These practices can help extend the life of the net since the net is less likely to be damaged if stored and less frequent washing results in longer lasting insecticide. These approaches can be customized to other contexts to promote care and repair to promote net integrity and ultimately lengthen useful net life.

The results showed that the availability of a new net was unlikely to be a trigger for discarding an existing net in decent condition. Most of the study respondents reported that if they were in a situation where they had a usable net and received a new net, they would use the new net but save the old net for later or share it with family, friends, or neighbours. This shows that in Senegal, nets are highly valued and that extra nets in households perceived as still useable are likely being used or shared rather than discarded. This suggests that an over-supply of nets within a given household would likely be used or redistributed to family or friends without access to a net. Interestingly, in Louga, respondents mentioned that they would prefer to continue using the old net until it was no longer possible. At this point, they suggested that they could switch to the new net. It is unclear why this was the case in Louga, but it may be a function of a different net culture or of the region having recently received nets through a mass campaign. It is possible that people in this region had been exposed to making such a decision more recently than others.

While it is important that people care for and repair their nets to prolong net life, nets will ultimately become too damaged to use and will need to be replaced. Since no universally accepted definition of the end of life of a net exists and responses varied on the end of a net’s life, guidelines for determining the appropriate end of net life should be developed and communicated to net users. Net users need to have an increased understanding of how long nets are expected to last, how best to care for and repair the net, and at what point the net is no longer protecting them from malaria.

Continuous distribution of LLINs has become an increasingly important component of vector control. These mechanisms, which help to achieve and maintain universal coverage, can include distribution through health facilities via antenatal care (ANC) consultations, schools, immunization campaigns and other channels to ensure long-term, routine access to nets [37,38]. Robust continuous distribution systems are essential so that people have the opportunity to obtain a new net when their old net expires and avoid putting themselves at increased risk of malaria. In order to avoid a coverage gap, comprehensive guidance is needed to balance the priorities of extending net life for as long as possible and making replacement nets available through continuous distribution systems.

Existing research on willingness to pay for nets shows that this is largely dependent on one’s socio-economic status [39]. Those of lower wealth are less willing and able to pay for nets in terms of both theoretical and actual behaviour [39]. Equity issues and considerations of the population’s willingness and ability to pay should be incorporated into the design of continuous distribution systems and subsidized net sales. This will allow all segments of the population to get a new net when they determine that their existing net has reached expired status.

The results of this research show that nets are highly valued in these communities and that often, nets that have reached the end of their useful life are repurposed for alternative uses. Alternative uses of nets have been documented in the published literature [19,22,28,40] but there has been mixed evidence on net condition at the time of repurposing. Participants in this study reported that most often expired rather than active nets are used by people in their communities for purposes such as protecting seedlings, covering meat in butcheries, filtering water and as curtains in windows. This finding supports other research that shows that it is primarily old nets that are used for other purposes [19,32]. However, it stands in contrast to other research that shows that both active and expired nets are repurposed and that in some cases, nets are even purchased for alternative use due to the high value of the material [22,29]. These results are consistent with data from Timor-Leste [22] in that participants are more likely to talk about alternative net use among others in their communities rather than their own behaviour. This reliance on hearsay may indicate that the repurposing of nets is a sensitive topic in communities; this is consistent with other research which shows that community leaders sometimes impose fines for the misuse of nets [19]. The results of this study demonstrate the very high value placed on nets in Senegal, even when they have reached the end of their useful life, paralleling findings in Madagascar [28]. As in Madagascar, the high value of nets and preference for repurposing are likely to make a net collection or net recycling programme unsuccessful and unnecessary [28].

As noted in the results, several of the alternative uses, such as protecting plants from insects and use at a butchery to keep flies off of meat, may be dependent upon having remnant insecticide. Even when torn, nets with active insecticide can be useful for malaria prevention and are likely superior to sleeping without a net [41]. This message, along with guidelines around useful net life, should be communicated to communities so that nets with active insecticide are prioritized for sleeping.

These results showed both regional and urban/peri-urban variation regarding alternative net use. Participants in Dakar, Thies and Kedougou reported that they were not aware of alternative uses of nets in their communities. This may have been due to a lack of excess nets in these regions as Dakar and Thies had not yet received nets through the universal coverage campaign and Kedougou had received nets during the first phase of distribution in 2010. Dakar and Thies are more urban regions and may not have the same needs for net repurposing as other regions. Additional research is needed to examine the differences in repurposing behaviour regionally and the factors motivating this behaviour.

### Limitations

The research conducted was qualitative in nature, and was therefore meant to reflect the in-depth experiences of a small number of respondents and cannot be applied to other context. While the sample was purposively selected to reflect the experiences of people in different regions throughout Senegal, these respondents were not necessarily representative of others in their community. However, the results of this study do reflect the experiences and perceptions of net owners in Senegal and can be used programmatically for the development of health communication messages.

The research was designed to include projective techniques, where respondents reflected on and responded to hypothetical situations rather than reporting on their own behaviour. This was done in order to reduce any stigma associated with throwing away or repurposing one’s own net. It also allowed for the standardization of responses so that all respondents were discussing the same situation rather than reflecting on their own experiences. However, the use of theoretical situations may have been challenging for respondents and should be complemented with personal experiences in future studies.

The nets used in the second scenario were all collected from the fieldworker’s homes so that they would have some signs of use. Once collected, they were torn by the field team to include between ten and 15 large holes. While the study team aimed to make the nets used in each region as comparable as possible, this process was left to the discretion of members of the field team rather than using objective criteria. Therefore, the responses from participants in the various regions may reflect some of the minor differences in the net conditions.

Finally, in Louga and perhaps in other regions, respondents thought that the data collectors were involved in the net distribution and thanked them for nets. The data collectors informed them that the research team was independent from the net distribution and from the government. However, it is possible that some respondents may have thought that the data collection team was related to the net distribution, thereby biasing results.

## Conclusions

In the context of uncertain resources, it is important that households continue to use their nets for as long as possible. The lengthening of net life also maximizes the value for money of nets procured by individuals, governments, and aid donors. This research provides an initial look into the cues for retiring or repurposing one’s net. Additional research is needed to further understand how households decide to stop using nets and how health communication programmes can encourage communities to undertake measures to prolong useful net life and prevent damage through care and repair activities. Finally, malaria policymakers need to develop recommendations surrounding the useful life of nets and communicate these guidelines to those in malaria-endemic countries, while implementing strong continuous distribution systems to ensure access to replacement nets, as needed. This will help to ensure that people are protected by their nets for as long as possible, without putting themselves at risk by continuing to use nets that are no longer protective.

## Competing interests

The authors declare that they have no competing interests.

## Authors’ contributions

DL participated in the study and instrument design, oversaw fieldworker training and fieldwork, analysed the data and drafted the manuscript. SB participated in the fieldworker training prior to data collection and provided in-depth review and edit of the manuscript. IW and SF supervised and conducted data collection and preliminary analysis of results. HK provided in-depth review and edited the manuscript. BA assisted in data analysis. RW designed the study and instruments and edited the manuscript. All authors read and approved the final manuscript.

## References

[B1] Lengeler C Insecticide-treated bed nets and curtains for preventing malaria Cochrane Database Syst Rev 2004 2 CD000363 15106149 10.1002/14651858.CD000363.pub2

[B2] WHO World Malaria Report 2012 Geneva: World Health Organization

[B3] Allan R O'Reilly L Gilbos V Kilian A An observational study of material durability of three world health organization–recommended long-lasting insecticidal nets in eastern Chad Am J Trop Med Hyg 2012 87 407 10.4269/ajtmh.2012.11-0331 22802441 PMC3435340

[B4] Kilian A Byamukama W Pigeon O Gimnig J Atieli F Koekemoer L Protopopoff N Evidence for a useful life of more than three years for a polyester-based long-lasting insecticidal mosquito net in Western Uganda Malar J 2011 10 299 10.1186/1475-2875-10-299 21992483 PMC3212829

[B5] Stevens ER Aldridge A Degbey Y Pignandi A Dorkenoo MA Hugelen-Padin J Evaluation of the 2011 long-lasting, insecticide-treated net distribution for universal coverage in Togo Malar J 2013 12 162 10.1186/1475-2875-12-162 23680434 PMC3658896

[B6] West PA Protopopoff N Rowland MW Kirby MJ Oxborough RM Mosha FW Malima R Kleinschmidt I Evaluation of a national universal coverage campaign of long-lasting insecticidal nets in a rural district in north-west Tanzania Malar J 2012 11 273 10.1186/1475-2875-11-273 22882836 PMC3465191

[B7] Ye Y Patton E Kilian A Dovey S Eckert E Can universal insecticide-treated net campaigns achieve equity in coverage and use? The case of northern Nigeria Malar J 2012 11 32 10.1186/1475-2875-11-32 22297189 PMC3312823

[B8] Pulford J Hetzel MW Bryant M Siba PM Mueller I Reported reasons for not using a mosquito net when one is available: a review of the published literature Malar J 2011 10 83 10.1186/1475-2875-10-83 21477376 PMC3080352

[B9] Atkinson J-A Bobogare A Fitzgerald L Boaz L Appleyard B Toaliu H Vallely A A qualitative study on the acceptability and preference of three types of long-lasting insecticide-treated bed nets in Solomon Islands: implications for malaria elimination Malar J 2009 8 119 10.1186/1475-2875-8-119 19497127 PMC2699345

[B10] Winch PJ Makemba AM Kamazima S Lurie M Lwihula G Premji Z Minjas JN Shiff CJ Local terminology for febrile illnesses in Bagamoyo District, Tanzania and its impact on the design of a community-based malaria control programme Soc Sci Med 1996 42 1057 1067 10.1016/0277-9536(95)00293-6 8730911

[B11] Adongo PB Kirkwood B Kendall C How local community knowledge about malaria affects insecticide-treated net use in northern Ghana Trop Med Int Health 2005 10 366 378 10.1111/j.1365-3156.2005.01361.x 15807801

[B12] Toé LP Skovmand O Dabiré KR Diabaté A Diallo Y Guiguemdé TR Doannio J Akogbeto M Baldet T Gruénais M-E Decreased motivation in the use of insecticide-treated nets in a malaria endemic area in Burkina Faso Malar J 2009 8 175 10.1186/1475-2875-8-175 19640290 PMC2729312

[B13] Okrah J Traore C Palé A Sommerfeld J Müller O Community factors associated with malaria prevention by mosquito nets: an exploratory study in rural Burkina Faso Trop Med Int Health 2002 7 240 248 10.1046/j.1365-3156.2002.00856.x 11903986

[B14] Baume CA Marin MC Intra-household mosquito net use in Ethiopia, Ghana, Mali, Nigeria, Senegal, and Zambia: are nets being used? Who in the household uses them? Am J Trop Med Hyg 2007 77 963 971 17984361

[B15] Baume CA Reithinger R Woldehanna S Factors associated with use and non-use of mosquito nets owned in Oromia and Amhara regional states, Ethiopia Malar J 2009 8 264 10.1186/1475-2875-8-264 19930654 PMC2796673

[B16] Pulford J Oakiva T Angwin A Bryant M Mueller I Hetzel MW “Indifferent to disease”: A qualitative investigation of the reasons why some Papua New Guineans who own mosquito nets choose not to use them Soc Sci Med 2012 75 2283 2290 10.1016/j.socscimed.2012.08.030 22995668

[B17] Beer N Ali AS Eskilsson H Jansson A Abdul-Kadir FM Rotllant-Estelrich G Abass AK Wabwire-Mangen F Björkman A Källander K A qualitative study on caretakers' perceived need of bed-nets after reduced malaria transmission in Zanzibar, Tanzania BMC Public Health 2012 12 606 10.1186/1471-2458-12-606 22863188 PMC3438043

[B18] Bauch JA Gu JJ Msellem M Mårtensson A Ali AS Gosling R Baltzell KA Perception of malaria risk in a setting of reduced malaria transmission: a qualitative study in Zanzibar Malar J 2013 12 75 10.1186/1475-2875-12-75 23433302 PMC3584900

[B19] Koenker HM Loll D Rweyemamu D Ali AS A good night's sleep and the habit of net use: perceptions of risk and reasons for bed net use in Bukoba and Zanzibar Malar J 2013 12 203 10.1186/1475-2875-12-203 23764006 PMC3691710

[B20] Alaii JA Hawley WA Kolczak MS Kuile FO Gimnig JE Vulule JM Odhacha A Oloo AJ Nahlen BL Phillips-Howard PA Factors affecting use of permethrin-treated bed nets during a randomized controlled trial in western Kenya Am J Trop Med Hyg 2003 68 137 141 12749497

[B21] Dunn CE Le Mare A Makungu C Malaria risk behaviours, socio-cultural practices and rural livelihoods in southern Tanzania: implications for bednet usage Soc Sci Med 2011 72 408 417 10.1016/j.socscimed.2010.11.009 21211875

[B22] Lover AA Sutton BA Asy AJ Wilder-Smith A An exploratory study of treated-bed nets in Timor-Leste: patterns of intended and alternative usage Malar J 2011 10 199 10.1186/1475-2875-10-199 21777415 PMC3155971

[B23] Grietens KP Ribera JM Soto V Tenorio A Hoibak S Aguirre AR Toomer E Rodriguez H Cuentas AL D'Alessandro U Traditional nets interfere with the uptake of long-lasting insecticidal nets in the Peruvian Amazon: the relevance of net preference for achieving high coverage and use PLoS One 2013 8 e50294 10.1371/journal.pone.0050294 23300943 PMC3534704

[B24] Pigott DM Atun R Moyes CL Hay SI Gething PW Funding for malaria control 2006–2010: A comprehensive global assessment Malar J 2012 11 1 11 10.1186/1475-2875-11-1 22839432 PMC3444429

[B25] Leach-Kemon K Chou DP Schneider MT Tardif A Dieleman JL Brooks BP Hanlon M Murray CJ The global financial crisis has led to a slowdown in growth of funding to improve health in many developing countries Health Aff 2012 31 228 235 10.1377/hlthaff.2011.1154 22174301

[B26] Kisinza WN Mutalamwa PP Kisoka WJ Munga MA Bilali K Masue D Social, cultural and ethical issues related to the life cycle management of LLINs: A case study of Mtwara rural, Kilombero, and Muheza districts 2011 National Institute for Medical Research, Amani Mediacl Research Centre

[B27] Ngondi JM Graves PM Gebre T Mosher AW Shargie EB Emerson PM Richards FO Jr Which nets are being used: factors associated with mosquito net use in Amhara, Oromia and Southern Nations, Nationalities and Peoples’ Regions of Ethiopia Malar J 2011 10 92 10.1186/1475-2875-10-92 21496331 PMC3107818

[B28] Ramanantsoa A Rahenintsoa R Hoibak S Ranaivoharimina H Rahelimalala MD Rakotomanga A Finlay A Ribera JM Hausmann-Muela S Toomer E Can the recycling of LLIN reduce their coverage and use? Social, cultural and ethical aspects of LLIN life cycle management: exploratory qualitative data from Madagascar Malar J 2012 11 77 10.1186/1475-2875-11-77 22439637 PMC3338400

[B29] Hoiback S Personal communication 2011

[B30] MSP Distribution gratuite de moustiquaires couplee aux journees locales de supplementation en viatmine a et deparasitage entre juin et octobre 2009 2011

[B31] NetWorks Senegal website 2013 http://networkssenegal.org

[B32] Mutuku FM Khambira M Bisanzio D Mungai P Mwanzo I Muchiri EM King CH Kitron U Physical condition and maintenance of mosquito bed nets in Kwale County, coastal Kenya Malar J 2013 12 1 14 10.1186/1475-2875-12-1 23374429 PMC3572415

[B33] Norris LC Norris DE Efficacy of long-lasting insecticidal nets in use in Macha, Zambia, against the local Anopheles arabiensis population Malar J 2011 10 254 10.1186/1475-2875-10-254 21880143 PMC3175477

[B34] Panter-Brick C Clarke SE Lomas H Pinder M Lindsay SW Culturally compelling strategies for behaviour change: a social ecology model and case study in malaria prevention Soc Sci Med 2006 62 2810 2825 10.1016/j.socscimed.2005.10.009 16352385

[B35] Harvey SA Olórtegui MP Leontsini E Asayag CR Scott K Winch PJ Trials of improved practices (TIPs): a strategy for making long-lasting nets last longer? Am J Trop Med Hyg 2013 88 1109 1115 10.4269/ajtmh.12-0641 23530074 PMC3752810

[B36] Widmar M Nagel CJ Ho DY Benziger PW Hennig N Determining and addressing obstacles to the effective use of long-lasting insecticide-impregnated nets in rural Tanzania Malar J 2009 8 315 10.1186/1475-2875-8-315 20043830 PMC2809069

[B37] Grabowsky M Nobiya T Selanikio J Sustained high coverage of insecticide**-**treated bednets through combined Catch**-**up and Keep**-**up strategies Trop Med Int Health 2007 12 815 822 10.1111/j.1365-3156.2007.01862.x 17596247

[B38] Kilian A Wijayanandana N Ssekitoleeko J Review of delivery strategies for insecticide treated mosquito nets: are we ready for the next phase of malaria control efforts? TropIKA Net 2010 1 1 28

[B39] Onwujekwe O Hanson K Fox-Rushby J Inequalities in purchase of mosquito nets and willingness to pay for insecticide-treated nets in Nigeria: challenges for malaria control interventions Malar J 2004 3 6 10.1186/1475-2875-3-6 15023234 PMC395839

[B40] Minakawa N Dida GO Sonye GO Futami K Kaneko S Unforeseen misuses of bed nets in fishing villages along Lake Victoria Malar J 2008 7 58 10.1186/1475-2875-7-58 18752662 PMC2532690

[B41] Malima RC Magesa SM Tungu PK Mwingira V Magogo FS Sudi W Mosha FW Curtis CF Maxwell C Rowland M An experimental hut evaluation of Olyset nets against anopheline mosquitoes after seven years use in Tanzanian villages Malar J 2008 7 6 10.1186/1475-2875-7-6 18307802 PMC2267806

